# Therapeutic Values of Human Urinary Kallidinogenase on Cerebrovascular Diseases

**DOI:** 10.3389/fneur.2018.00403

**Published:** 2018-06-05

**Authors:** Zhenyu Wei, Yi Lyu, XiaoLi Yang, Xin Chen, Ping Zhong, Danhong Wu

**Affiliations:** ^1^Department of Neurology, Shanghai Fifth People's Hospital, Fudan University, Shanghai, China; ^2^Department of Medical Affairs, Techpool Bio-Pharma Co. Ltd., Guangzhou, China; ^3^Department of Neurology, Shanghai TCM Integrated Hospital affiliated to Shanghai University of Chinese Medicine, Shanghai, China

**Keywords:** acute ischemic stroke, angiogenesis, neurogenesis, inflammation, human urinary kallidinogenase

## Abstract

The term “tissue kallikrein” is used to describe a group of serine proteases shared considerable sequence homology and colocalize in the same chromosomal locus 19q13. 2–q13.4. It has been widely discovered in various tissues and has been proved to be involved in kinds of pathophysiological processes, such as inhibiting oxidative stress, inflammation, apoptosis, fibrosis and promoting angiogenesis, and neurogenesis. Human Urinary Kallidinogenase (HUK) extracted from human urine is a member of tissue kallikrein which could convert kininogen to kinin and hence improve the plasma kinin level. Medical value of HUK has been widely investigated in China, especially on acute ischemic stroke. In this review, we will summarize the therapeutic values of Human Urinary Kallidinogenase on acute ischemic stroke and its potential mechanisms.

## Introduction

The term “tissue kallikrein” is used to describe a series of acid glycoprotein with highly similar gene and protein structure, which also share considerable sequence homology and colocalize in the same chromosomal locus 19q13.2–q13.4 (1, 2). Tissue Kallikrein is encoded by one of the kallikrein gene family, KLK1 gene. Originally, it was thought that the gene family included three genes, namely prostate-specific antigen, tissue kallikrein, and glandular kallikrein 2 (3). In 2000, other 12 kallikrein-related genes were found, and, until now, 15 tissue kallikrein genes have been discovered (4).

Tissue kallikreins release vasoactive peptides from low molecular weight kininogen. The peptides, mainly including kinin, trigger multiple biological functions by activating two types of G protein-coupled receptors named as B1 receptor and B2 receptor respectively (5). Tissue kallikrein is widely distributed such as in kidney, blood vessels, central nervous system, pancreas, gut, neutrophils, and so on (6). HUK is one of the tissue kallikreins that has been found in human urine. Urine product has been believed to have medical value for centuries. Galen, Greek physicians used urine product to cure inflammation, burns and skin disease, and in Japan, urine product was used to cure asthma, hypertension, and diabetes (7). The potential therapeutic value of urine reminds us that tissue kallikrein may also be expected to have therapeutic effects. But previous studies found that protective role of tissue kallikrein does not work on all types of hypertension, and that tissue kallikrein is expected to be useful for treatment of salt-sensitive hypertension (8, 9). Now in China, HUK has been used for acute ischemic stroke and recent studies has found that HUK also exert multiple functions such as attenuating brain injury by inhibiting oxidative stress and promoting angiogenesis and neurogenesis. HUK also has clinical application on cardiac and renal damage, vascular restenosis, ischemic stroke, skin wound injury, and hypertension. In this review, we summarized therapeutic values of HUK on cerebrovascular diseases by the results from clinical trials and animal experiments.

## Result from clinical trials

### HUK improves neurological function of patients with ischemic stroke

In a case–control study with 1,268 stroke patients, plasma tissue kallikrein levels were negatively related to the risk of first-ever stroke and stroke recurrence, which suggested that lower plasma tissue kallikrein levels are independently correlated to first-ever stroke and are an independent predictive factor of stroke recurrence (10). Tissue kallikrein is more likely involved to the pathological process of stroke. In ischemic stroke, HUK application significantly decreases National Institute of Health Stroke Scale (NIHSS) and modified Rankin Scale (mRS) scores and improves long-term outcomes (11, 12). It was discovered that HUK was able to significantly improve the NIHSS scores of cerebral ischemia resulted from large-artery atherosclerosis and small-artery atherosclerosis, the results from the study on the clinical efficacy of HUK in the treatment of ischemic stroke according to TOAST classification (13). In the studies of combined medicine, HUK may improve the curative effect of rt-PA in patients with acute ischemic stroke (AIS) (14), and improve therapeutic effect of massive cerebral infarction when combined with edaravone (15).

After treatment with HUK, AIS patents' cerebral blood perfusion was significantly enhanced and mean transit time of perfusion was remarkably shorten, which are in accordance with increasing serum levels of VEGF and apelin (16). These two substances are all involved in vascular formation and maturation (17, 18). In a small sample clinical research performed in acute ischemic stroke patients treated with HUK or not, microcirculation was evaluated by magnetic resonance perfusion weighted imaging (MRP). It showed HUK could increase cerebral blood flow and ameliorate neurological deficits (19). Moreover, Wang et al found that HUK remarkably increased ipsilesional sensorimotor cortex (SMC) blood volume in AIS patients (11). The effect of HUK on promoting cerebral reorganization might be an critical mechanism in the treatment of acute cerebral infarction. In a small sample research, Song et al. discovered that Human Urinary Kallidinogenase improved symptoms of neurological dysfunction by promoting remodeling of long-term cortical motor function in patients with ischemic stroke as well (20).

### HUK prevent cerebrovascular restenosis after endovascular therapy

The endovascular therapy study enrolled two groups of patients with symptomatic middle cerebral artery stenosis. Among them, HUK intervention was administered in one group and the other group as control. Carefully follow-up and evaluation for restenosis showed HUK could prevent restenosis after in-stent in middle cerebral artery (21, 22). For patients with cerebral arterial stenosis and without stroke treated by HUK, cognitive status could be improved accompanied by Aβ1-40 serum levels decreasing at 4 weeks after treatment and Aβ1-42 serum levels decreasing remarkably at 8 weeks after treatment (23). The potential mechanism of preventing restenosis was to inhibit atherosclerosis formation and to attenuate intimal hyperplasia by downregulating Smad2/3 phosphorylation and TGF-β1 expression, promoting eNOS activity (24).

### Safety assessment of human urinary kallidinogenase

To assess the safety of HUK in treating patients with acute ischemic stroke, a systematic review on randomized controlled clinic trials about HUK has been performed, and all the data has been gotten from the Chinese Stroke Trials Register, the Cochrane Stroke Group Trials Register, CENTRAL, Medline, EMBASE, the China Biological Medicine Database (CBM), and the China National Knowledge Infrastructure (CNKI) (12). The research included 24 trials involving 2433 patients, in which 15 trials reported that the common adverse event was transient hypotension, and there was no difference between the treatment group and control group on non-fatal intracerebral hemorrhage. A multi-center, open label, single group study has been designed and registered in ClinicalTrials.gov to reevaluate the safety and efficacy of HUK on acute ischemic stroke, and the study expects to enroll 60 sites and 2186 subjects (25).

## Results from animal experiments

### Angiogenesis and enhanced neurogenesis

It has been proved that HUK promoted angiogenesis and enhanced neurogenesis after ischemia/reperfusion (I/R) induced injury (26). Another animal experiment demonstrates that HUK treatment significantly increased the number of BrdU(+) cells in the subventricular zone (SVZ) and the peri-infarction region at 3 d after middle cerebral artery occlusion (MCAO), noteworthily, the number of BrdU(+)/DCX(+) cells and BrdU(+)/nestin(+) cells in the SVZ as well as vascular density in the peri-infarction region increased more significantly than vehicle group at 3 d, peaked at 7–14 d. HUK increased the number of BrdU(+)/NeuN(+) cells in the peri-infarction region at 14 and 28 d after MCAO compared with the vehicle group (27). However, in an animal experiment study about choroidal neovascularization (CNV), pretreatment with HUK could significantly decrease laser-induced CNV size (28).

### Inflammation and apoptosis

A continuous infusion of a sub-depressor dose of HUK by minipump decreased I/R-induced neurological disorders and brain infarction, inflammation, and oxidative stress, which suggested that HUK also acted as an anti-oxidative and anti-inflammatory agent in protecting the brain from ischemic stroke induced damages (29). Yang et al. found that HUK improves neurological deficits, reduces the infarct volume, and decreases inflammatory responses in rats following cerebral ischemia. Meanwhile, they also found that the levels of TLR4 and NF-kB in ischemic brains increased following MCAO by western blot analysis, and this effect was significantly inhibited by immediate treatment of HUK (30). It concluded that tissue kallikrein protects against ischemic stroke through antioxidation and anti-inflammation by suppressing TLR4/NF-κB signaling pathway in rats.

Transforming growth factor beta 1 (TGF-β1), as an immunomodulator, can attenuate inflammation definitely, and previous studies confirmed that TGF-β1 limited neuroinflammation and that activated the expression of Bcl-2 to suppress the apoptosis of neurons in acute ischemic stroke rats (31, 32). HUK treatment can highly increase the expression of TGF-β1 in rats with focal ischemic injury, meanwhile, decrease high-sensitivity c-reactive protein expression, a systemic inflammation indicator (33). Glutamate-induced neurotoxicity induced oxidative injury and was involved in the pathogenesis of a series of central nervous system disorders. Previous studies found that HUK could largely inhibit glutamate-induced cell death and morphological changes of cultured cortical neurons by inhibiting of ROS production and attenuating nNOS activity to inhibit glutamate-induced nitrosative stress through an intracellular signaling pathway including activation of B2R, ERK1/2, and NFkB, and up-regulation of BDNF and Bcl-2 expression (34–36). Jingjing Su et al. demonstrated that HUK could activate ERK1/2 signaling cascades through Homer1b/c (37). Xia et al. proved that local or systemic delivery of the tissue kallikrein gene attenuates cerebral I/R injury in mouse models by inhibiting oxidative stress and apoptosis through activation of B2R (26). Besides, B2R-dependent regulation of autophagy is involved in inhibiting oxygen and glucose deprivation-induced neuronal cells injury (38).

Except for neuroprotective effects, it has been demonstrated that HUK might prevent and ameliorate diabetes-induced nephropathy and reduced renal inflammation, oxidative stress and renal fibrosis (39). The activation of the kallikrein-kinin system was also found in the experiment. However, some evidences suggested that kinins may play an important role in the development of diabetic retinopathy by enhancing vascular permeability, leukocytes infiltration, and inflammatory response through mediating kinin B1 and B2 receptors (40).

## Conclusions

Recent animal experiments and clinic trials proved that HUK has enormous therapeutic value in acute ischemic stroke. In patients treated with HUK, the enhanced microcirculation and cerebral blood flow may promote angiogenesis and neurogenesis and inhibit inflammation and apoptosis. However, recent optimal results were from small sample clinical trials, the mechanism of HUK has not been clear enough, evidences of the safety with HUK are still absence, more animal experiments and multicenter-clinical trials are necessary in the future.

## Author contributions

ZW and YL prepared the references and designed the structure of this paper. ZW, XY, and XC prepared the manuscript. PZ and DW reviewed and revised this article.

### Conflict of interest statement

YL was employed by company Techpool Bio-Pharma Co.,Ltd. The remaining authors declare that the research was conducted in the absence of any commercial or financial relationships that could be construed as a potential conflict of interest.
